# Mitotic kinases are emerging therapeutic targets against metastatic breast cancer

**DOI:** 10.1186/s13008-024-00125-x

**Published:** 2024-06-17

**Authors:** Alexandra N. Aquino-Acevedo, Joel A. Orengo-Orengo, Melanie E. Cruz-Robles, Harold I. Saavedra

**Affiliations:** Department of Basic Sciences, Ponce Health Sciences University-Ponce Research Institute, 388 Luis Salas Zona Industrial Reparada 2, P.O. Box 7004, Ponce, Puerto Rico 00716-2347 USA

**Keywords:** Centrosome, Mitosis, The spindle assembly checkpoint, Aurora kinases a and B, TTK, NEK2, PLK1, Triple-negative breast cancer, Mitotic kinase inhibitors

## Abstract

This review aims to outline mitotic kinase inhibitors’ roles as potential therapeutic targets and assess their suitability as a stand-alone clinical therapy or in combination with standard treatments for advanced-stage solid tumors, including triple-negative breast cancer (TNBC). Breast cancer poses a significant global health risk, with TNBC standing out as the most aggressive subtype. Comprehending the role of mitosis is crucial for understanding how TNBC advances from a solid tumor to metastasis. Chemotherapy is the primary treatment used to treat TNBC. Some types of chemotherapeutic agents target cells in mitosis, thus highlighting the need to comprehend the molecular mechanisms governing mitosis in cancer. This understanding is essential for devising targeted therapies to disrupt these mitotic processes, prevent or treat metastasis, and improve patient outcomes. Mitotic kinases like Aurora kinase A, Aurora Kinase B, never in mitosis gene A-related kinase 2, Threonine-Tyrosine kinase, and Polo-kinase 1 significantly impact cell cycle progression by contributing to chromosome separation and centrosome homeostasis. When these kinases go awry, they can trigger chromosome instability, increase cell proliferation, and activate different molecular pathways that culminate in a transition from epithelial to mesenchymal cells. Ongoing clinical trials investigate various mitotic kinase inhibitors as potential biological treatments against advanced solid tumors. While clinical trials against mitotic kinases have shown some promise in the clinic, more investigation is necessary, since they induce severe adverse effects, particularly affecting the hematopoietic system.

## Background

For the past three decades, breast cancer incidence and mortality rates have increased among women [1]. This represents a serious global health threat. Breast cancer can be divided into several subtypes, based on the expression levels of specific receptors; these levels dictate the therapies that are used to treat a patient [2]. For example, triple-negative breast cancer (TNBC) is an aggressive subtype of breast cancer characterized by the absence of the estrogen receptor (ER), progesterone receptor (PR) expression, and human epidermal growth factor receptor 2 (HER2) amplification [3, 4]. The absence of these receptors renders TNBC insensitive to both hormone therapy and HER2-targeted therapies, making chemotherapy the primary treatment option [5], therefore limiting the therapeutic options for TNBC [6]. While TNBC is considered the most aggressive subtype of breast cancer, there are no known effective targeted therapies to date. Two novel biological therapies are emerging against TNBC. One is immunotherapy, which can be effective in 20% of patients who overexpress high levels of PD-L1 in their TNBCs [7, 8]. Another one is anti-mitotic therapy, which we discuss here. The aggressive nature of TNBC is linked to its increased propensity for relapse and unique patterns of metastasis, including visceral metastasis [9]. TNBC is associated with a higher risk of metastasis due to its inherent genetic instability and increased proportions of actively proliferating cells relative to other subtypes, which can lead to the propagation and accumulation of genetic mutations that drive invasive and metastatic behaviors [4, 10]. Cells replicate their DNA at S-phase and divide their DNA during mitosis, allowing tumors to grow [11, 12]. TNBC tumors often show a high Ki67-positive fraction, indicating a larger proportion of actively proliferating cells [13]. These factors likely contribute to the accumulation of genetic mutations that drive invasive and metastatic behaviors. Mitosis has been broadly studied in TNBC, as it plays a critical role in its progression due to its high proliferation rates [14, 15]. Disruptions in the regulation of mitosis can lead to abnormal chromosome segregation, resulting in aneuploidy and genomic instability [16–18]. Another cellular change that impinges on mitosis is centrosome amplification, or the acquisition of ≥ 3 centrosomes that can promote chromosome instability and aneuploidy [19–22]. Centrosome amplification is a feature that is commonly identified in most breast cancer tumors [23, 24]. Mounting data also suggests that not only does centrosome amplification strongly correlate with a TNBC subtype, but it can also promote a more metastatic disease, as well as chromosome instability and tumorigenesis [19, 23, 25, 26]. These phenomena can lead to more aggressive and chemo-resistant breast cancer [27]. Moreover, different dysregulated signaling pathways can promote centrosome amplification via mitotic kinases, such as AURKA, NEK2, and PLK1 [28]. Hence, this suggests that mitotic kinases play a pivotal role in the process of centrosome amplification and that their overexpression can promote dysregulation in the cell cycle, leading to uncontrolled cell growth. These mitotic errors can drive tumor progression and contribute to the increased metastatic potential of TNBC cells. Therefore, understanding the molecular mechanisms that govern mitosis in TNBC is vital for developing targeted therapies that can disrupt these processes, prevent metastasis, and improve patient outcomes. Emerging experimental evidence suggests a role for mitotic kinases and signaling pathways that regulate the expression of these kinases in metastasis since they can drive early stages of metastasis, including the epithelial-to-mesenchymal transition, cell migration, and invasion [15, 29]. Mitotic kinases such as Aurora Kinase A (AURKA) and Aurora Kinase B (AURKB), Never in mitosis gene A-related kinase 2 (NEK2), Threonine-Tyrosine kinase (TTK), and Polo-kinase 1 (PLK1) play a critical role in cell cycle progression by regulating faithful chromosome segregation [6, 15, 30]. However, their dysregulation can promote centrosome amplification, aberrant mitotic checkpoints, abnormal cytokinesis that result in chromosome instability, aneuploidy, and cell proliferation, as well as the activation of different molecular pathways involved in the process of epidermal-to-mesenchymal transition (EMT) [15, 18, 31–35]. A previous study by Rivera et al. (2019) identified by using the TCGA database that several mitotic and centrosome regulators, including mitotic kinases, are overexpressed in TNBC, and are more likely to be overexpressed in non-Hispanic Black women with breast cancer, including TTK, NEK2, PLK1, Cyclin B1, BUB1, Aurora Kinase A, Aurora Kinase B and NDC 80 (HEC1) [6]. When only TNBCs are considered, PLK1 and AURKB are overexpressed in Non-Hispanic Black women, while TTK and TBK1 are overexpressed in Non-Hispanic White patients. Therefore, mitotic kinases can drive the aggressive nature of TNBC in women of African heritage, who are more prone to develop and to die from this highly aggressive subtype of breast cancer relative to women of European ancestry [36–41].

FOXM1 has been widely studied in the context of cancer due to its role in cell proliferation, cell cycle progression, apoptosis, and other cellular processes [42]. Since FOXM1 has been associated with a network of genes, it is crucial to investigate how its dysregulation can promote these cellular changes that can eventually lead to different types of cancer, including breast cancer. A recent study has demonstrated that TNBCs overexpress FOXM1 and that inhibiting FOXM1 in MDA-MB-231 (TNBC cell lines) can suppress their growth [42]. Therefore, this suggests that FOXM1 has a critical role in promoting cell proliferation. However, since FOXM1 can form a network with different genes, it is essential to know how these genes might also be dysregulated and promote a more aggressive TNBC subtype. In a study by Radovich et al., they determined that FOXM1 can regulate 47 genes out of the 146 TNBC core genes, which include TTK, AURKA, NEK2, and PLK1 [43]. Therefore, these findings demonstrate the importance of FOXM1 as a major regulator of different genes that are known to be dysregulated in TNBC. Moreover, Yang et al. 2017, found that inhibiting AURKA and FOXM1 simultaneously can decrease tumorigenesis and proliferation in MDA-MB- 231 cell lines [44]. Thus, these findings open the possibility of studying these genes regulated by FOXM1 as a biomarker and potential therapeutic targets against TNBC. Another pathway driving metastasis is the Rb/E2F pathway [29, 45–48], which controls the gene expression of many centrosome and mitotic regulators, including NEK2, PLK4, SgoI, TTK, AURKA, and AURKB, as well as matrix metalloproteases [33, 47–51]. The silencing of E2F3 can significantly reduce metastasis in TNBC models of breast cancer, and tumor growth rates in a Her2 + model by decreasing rates of mitosis [34, 45]. 

**Fig. 1:**
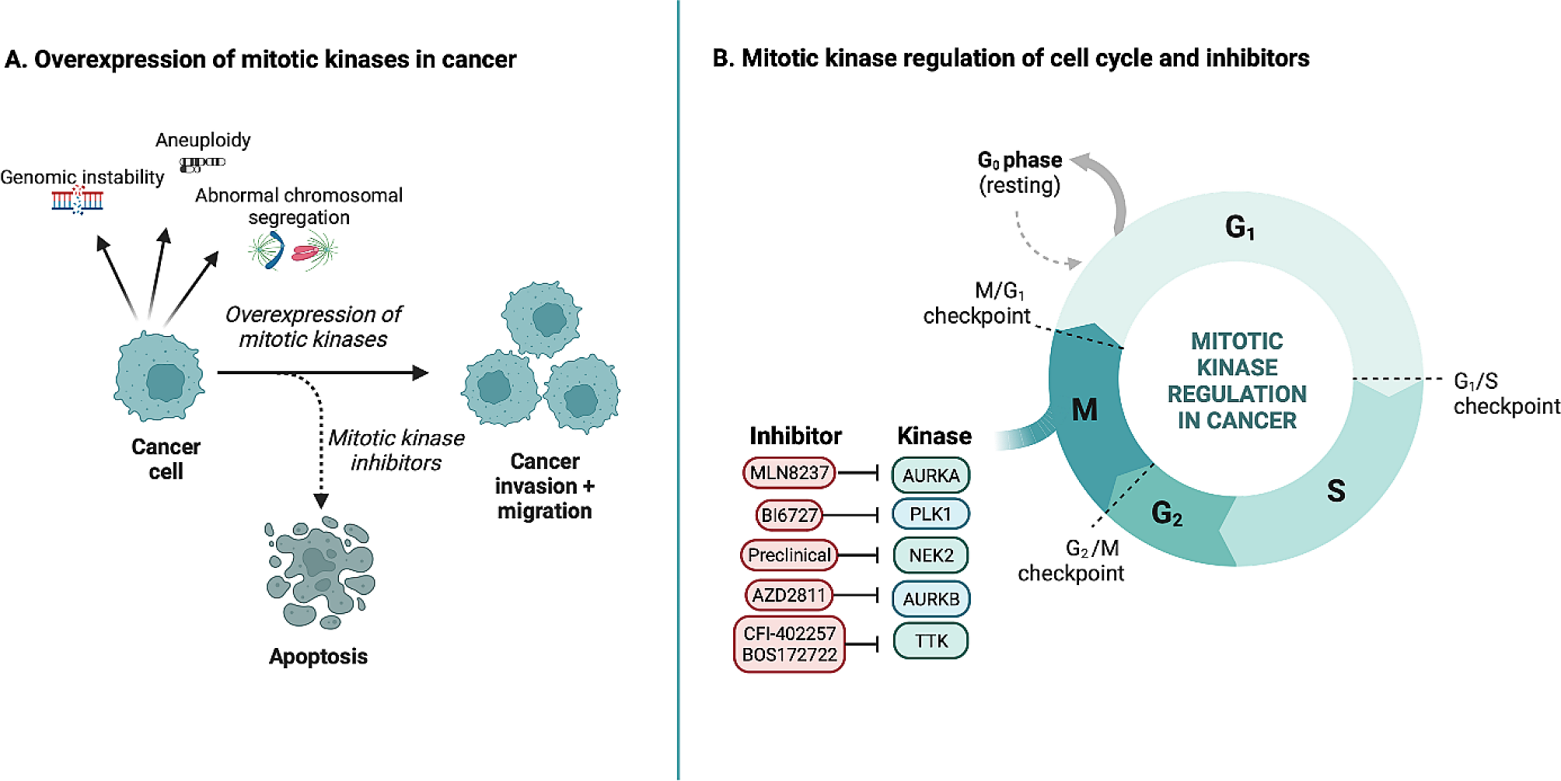
Summary of mitotic kinases overexpression (**A**) and inhibition (**B**) in the cell cycle of triple-negative breast cancer

Several mitotic kinase inhibitors are currently under clinical trials to evaluate their potential as treatments against advanced solid tumors, including TNBC. Although mitotic inhibitors, such as microtubule-targeting drugs like paclitaxel and vinorelbine, are commonly used in chemotherapy regimens for TNBC, drug resistance and side effects remain a significant challenge. TNBCs show paradoxical behavior towards chemotherapy (the TNBC paradox), since they respond better to chemotherapy than other subtypes, but also have a higher tendency to relapse and metastasize [52]. Thus, efforts to target mitosis as a novel therapeutic strategy in TNBC are ongoing [14]. In this review, we will discuss how the overexpression of mitotic kinases such as AURKA, AURKB, NEK2, TTK, and PLK1 are involved in different molecular pathways that contribute to the early stages of metastasis (EMT, cell migration, and cell invasion) of advanced-stage cancers, in particular breast cancer. Although these mitotic kinases show the potential to be targeted in breast cancer, their single inhibition may result in mitotic slippage, which grants resistance to cancer cells by potentially leading to chromosome instability [14]. This is one of the major concerns of using single mitotic kinase inhibitors, as they cannot induce a durable effect. As a result, most cancer cells can avoid the apoptotic pathway and develop resistance. Therefore, most current clinical trials are using combinations that involve a mitotic kinase inhibitor combined with other drugs, including anti-microtubule agents and an inhibitor that inhibits AURKA and pro-angiogenic kinases, or combinations that target AURKA with the anti-hormonal agent Fulvestrant [53–55]. We will detail these clinical trials below (Summarized in Fig. 1).

### Aurora Kinase A

Mitotic kinases such as AURKA and AURKB have gained much attention as potential therapeutic targets against cancer, due to their essential role in the cell cycle, especially during mitosis [56]. Previous research studies have uncovered that their dysregulation is implicated in several types of cancer, including breast cancer [6, 15]. AURKA is one of the mitotic kinases necessary for preparation for cell division. This protein is a serine/threonine mitotic kinase with multiple functions, including centrosome duplication, chromosome stability, and mitotic spindle formation [15, 57]. The centrosome is vital for different cellular processes such as cell division and cell morphology [58]. However, dysregulation of the centrosome cycle could lead to centrosome amplification, which can induce various cellular changes, such as chromosome instability and aneuploidy [20–22, 59]. Centrosome amplification also leads to important precursors to metastasis, including loss of cell polarity and cell invasion [60]. Because of these properties, targeting the different pathways that culminate in centrosome amplification has been proposed to be a novel therapeutic strategy [20]. Pathways that can drive centrosome amplification include those that impinge on G1/S phase regulators such as the Cdk2 and Cdk4 G1/S phase kinases, as well as centrosome and mitotic regulators, including AURKA, TTK, PLK1, and NEK2 [20, 32, 35, 59, 61–63]. It has been suggested that these dysregulations contribute to the aggressive behavior of more aggressive types of breast cancer, such as TNBC and Her2^+^ [35, 58, 64]. Because AURKA drives centrosome amplification, aneuploidy, and chromosome instability, when AURKA is dysregulated, it can function as an oncoprotein in breast cancer progression [57, 65, 66]. These properties have been associated with different molecular events that confer the cell’s ability to develop drug resistance and metastasize. AURKA is under the transcriptional control of the E2F3 transcription factor, which when dysregulated, mediates metastatic progression in TNBC [45, 50]. Further, studies have suggested that AURKA can transactivate the FOXM1 promoter in TNBC [67]. FOXM1 is a transcription factor crucial to the regulation of the cell cycle especially in mitosis [68]. FOXM1 is overexpressed in different subtypes of breast cancer including TNBC, and is necessary for mitotic progression and maintenance of chromosome stability [68]. Furthermore, the activation of FOXM1 has been associated with the development of cancers through the c-Myc oncogene [68]. The *c-Myc* gene is part of the Myc family and is crucial for cell cycle progression [69]. *c-Myc* is overexpressed in breast cancer [69]. In addition, it has been described that FOXM1 promotes chemoresistance in breast cancer while its inhibition restores trastuzumab response [68]. Moreover, knocking down the expression of AURKA decreases FOXM1 levels in TNBC cell lines, restoring the response to paclitaxel [67]. Therefore, these studies suggest an essential correlation between the overexpression of AURKA, which can lead to an upregulation in FOXM1 levels in TNBC cell lines. This may promote a more aggressive type of breast cancer and resistance to chemotherapy agents. Thus, while transcription factors are currently not therapeutic targets in the clinic, a potential approach to mitigate the effects of transcription factor dysregulation in breast cancers, is to target one or more mitotic kinases regulated by these transcription factors.

AURKA can also interact and phosphorylate BRCA1 and BRCA2 genes, which are known to promote breast cancer when deregulated [70]. A recent study suggests that *AURKA-HMMR*, a genetic variant of *AURKA*, is linked to increased breast cancer risk in *BRCA1* and *BRCA2* mutation carriers [71]. Additionally, overexpression of AURKA has been found in tumors with *BRCA1* and *BRCA2* mutations [72].

On the other hand, AURKA also interacts with the PI3K/Akt/mTOR signaling pathway. This interaction has been associated with multiple cancers, including breast cancer. This is due to the activation of different transcription factors that promote EMT [70]. For instance, the overexpression of PI3/Akt/mTOR is associated with the activation of Twist1 and Twist2, N-cadherin, and Snail1. These EMT factors can provide several advantages to the cancer cells, such as resistance to different chemotherapies [73]. Silencing the AKT2 pathway could also reduce Twist and suppress resistance to paclitaxel. Work by Xu et al. 2015 indicates how crucial the overexpression of AURKA is in conferring the cell the ability to be resistant to chemotherapy agents that are commonly used to treat cancer [73]. Further, this study demonstrated that overexpression of Snail1 can downregulate E-cadherin, CK8, and CK9 in breast cancer. Hence, overexpression of AURKA could lead to dysregulation in the PI3/Akt/mTOR pathway and an increase in EMT factors associated with more aggressive breast cancer. This suggests that even though these EMT transcription factors are highly dysregulated in breast cancer, targeting a more upstream protein such as AURKA might suppress progression to metastasis. Interestingly, a recent study demonstrated a high expression of Zeb1 in TNBC cell lines but removing this sole EMT factor did not reverse the EMT process [74]. Similarly, TNBC cell lines also have a high expression of Snail1, but its inactivation did not decrease EMT [75]. However, recent reports demonstrated that Zeb1 or Snail are required for cell invasion of TNBC cells [18, 45]. Due to the crucial functions of these proteins during the cell cycle and how their dysregulation can lead to cell proliferation, several mitotic kinase inhibitors have been tested as potential therapies against distinct types of cancer. Alisertib is one of the mitotic kinase inhibitors currently in clinical trials [14, 15]. Alisertib is an AURKA inhibitor that, by targeting AURKA, disrupts mitotic spindles and chromosome segregation, eventually leading to cell death [7]. Alisertib is a selective benzazepine against AURKA; therefore, it is more potent against AURKA than AURKB [76]. Clinical trials have demonstrated the efficacy of alisertib in targeting AURKA and its activity to decrease tumor growth. However, alisertib alone has also shown elevated levels of toxicity [14]. For that reason, recent clinical trials have included a combination of therapies to increase its effectiveness and decrease its toxicity. In combination therapy, a lower dose per drug is administered, to reduce toxic effects to the patients, while still delivering the drug’s anti-mitotic effects [14]. For instance, a Phase I clinical trial evaluated the combination of alisertib and fulvestrant in women resistant to aromatase inhibitor ER^+^ with metastatic breast cancer (NCT02860000) [55]. Remarkably, this clinical trial described that combining these two therapies showed promising results in increasing the median progression-free survival. The main severe (grade 4) adverse effects were neutropenia and hypertension. Another promising clinical trial involved metastatic ER^+^/HER2^−^ or TNBC patients treated with alisertib and paclitaxel (NCT01091428) [53]. This combination resulted in significantly improved progression-free survival of ER^+^PR^+^ and TNBC patients relative to paclitaxel alone. The main adverse events (grades 3 or 4) with paclitaxel plus alisertib vs. paclitaxel alone were neutropenia, anemia, diarrhea, and stomatitis or oral mucositis. A similar trial reported a trend toward improved progression-free survival; however, there was no significant response [77]. These outcomes demonstrate the potential of AURKA inhibitors combined with approved therapies against metastatic breast cancer.

### Aurora Kinase B

Aurora Kinase B is another mitotic kinase with multiple cell cycle functions. AURKB is a serine/threonine kinase that regulates the spindle assembly checkpoint and proper chromosome segregation [78]. This process is imperative to ensure that chromosomes attach to the spindle microtubules to complete the cell cycle. When kinetochores are not correctly attached, they send a checkpoint signal that inhibits the anaphase-promoting complex/cyclosome (APC/C) [79]. This signal stops the cycle to prevent unattached chromosomes from continuing to complete mitosis. AURKB is part of the chromosome passenger complex along with its substrates: INCENP, Borealin, and Survivin, which are necessary for properly attaching the microtubules and the spindle checkpoint [80]. Aurora kinase B helps place TTK, which is the first responder after detection of a missegregated chromosome, into centromeres, an event that activates the spindle assembly checkpoint (SAC) [81, 82]; on the other hand, TTK controls AURKB activity through the phosphorylation of Borealin [83]. When AURKB is overexpressed, it also increases the phosphorylation of Histone 3 (H3), which causes aneuploidy and chromosome instability as well as proliferation and metastasis [78].

Recent studies have evaluated how dysregulation in AURKB leads to increased phosphorylation of Survivin (an inhibitor of apoptosis) and its correlation with a worse prognosis in breast cancer [84]. These findings suggest that dysregulation in AURKB can suppress crucial cellular processes that ensure the propagation of cancer cells. Previous work in our laboratory has demonstrated that non-Hispanic Black women with TNBC have higher levels of AURKB, and PLK1 relative to non-Hispanic White women [6]. Another study demonstrated that knocking down the expression of AURKB and PLK1, which is a crucial mitotic kinase for the activation of AURKB, suppresses survivin activity [84]. This study also proposed that African American women have a dysregulation in mitotic kinases and, therefore, a more aggressive breast cancer than women with European ancestry.

AURKB is also involved in different molecular pathways when dysregulated. For example, one study showed that overexpression of this mitotic kinase can activate the PI3K/AKT signaling pathway in intrahepatic cholangiocarcinoma [85]. Activating this pathway can promote EMT by increasing the expression levels of N-cadherin and Vimentin. In breast cancer such as TNBC, AURKB overexpression can promote EMT by activating the AKT/mTOR signaling pathway [86]. AURKB overexpression can promote EMT by decreasing E-cadherin levels and increasing the levels of N-cadherin and Vimentin in TNBC cell lines. This is also supported by observations that overexpression of AURKB can promote the expression of and stabilize Snail1, an important EMT factor. By using an AURKB inhibitor, they demonstrated that blocking the expression of AURKB can decrease the expression of EMT factors such as Snail1; therefore, Zhang, J., et al., 2020 concluded that this mechanism can suppress metastasis [86].

AURKB inhibitors, such as AZD2811, are currently under clinical trials in patients with solid tumors, including breast cancer. In a Phase I clinical trial (NCT02579226), 51 patients were treated with AZD2811, of which 2% resulted in a partial response and 45.1% in stable disease [87]. Moreover, this study showed that AZD2811 was safe and tolerable in a 500 mg dose. Patients were also treated with Granulocyte Colony Stimulating Factor (G-CSF) to manage different toxicities such as neutropenia, a common and expected side effect of the treatment [87].

Studies have suggested combining therapies that could favor the apoptotic pathway to avoid the senescence state, which gives cancer cells the ability to be dormant and then restart growth phases and acquire resistance to therapies. Therefore, current research has focused on targeting different mitotic kinases to improve their effect against several types of cancer. Recently, a computational study has identified other molecules that can target all three Aurora Kinases (AURKA, AURKB, and AURKC) [88]. That report explored how these mitotic kinases have similar residues in their Adenosine triphosphate (ATP) binding site, which may help develop a novel therapy that could potentially target all the AURKs [88]. Because it has been suggested that overexpression of AURKA and AURKB is related to a more aggressive type of cancer such as TNBC, identifying molecules that could target the AURKs is a promising step toward developing novel therapies.

### TTK

The Threonine-Tyrosine kinase (TTK), also known as monopolar spindle 1 (Mps1), is a mitotic kinase that plays a pivotal role in the regulation of mitosis [89]. TTK is responsible for controlling the attachment and alignment of chromosomes to the mitotic spindle and ensuring the accuracy of chromosome segregation, a critical event in cell division [89]. TTK also ensures the detection and correction of errors in chromosome attachment, a process known as the spindle assembly checkpoint (SAC) [4]. This function is critical in preventing aneuploidy, a condition where cells have an abnormal number of chromosomes, often associated with cancer. As described above, TTK’s centromere localization is controlled by AURKB. Once in the centromere, TTK phosphorylates KNL1. TTK helps recruit the SAC components, BubR1:Bub3 and Mad1/Mad2, which bind and inhibit APC/Cdc20 (together called the mitotic checkpoint complex or MCC) to prevent progression through mitosis until kinetochores are properly aligned [81, 90]. Once all kinetochores have been captured by spindle microtubules, the MCC is disassembled and cells continue through mitosis and cytokinesis.

However, dysregulation of TTK can lead to chromosome missegregation, ultimately resulting in aneuploidy, a hallmark of many cancer types. TTK expression levels have been positively correlated with p53 mutations as well as poor survival [91]. Data suggests that high levels of TTK mRNA can protect breast cancer cells from aneuploidy, ensuring their proper chromosome segregation [17]. High levels of TTK could prevent existing aneuploid cancer cells from further gaining or losing chromosomes, preventing cancer cells from compromising their viability [10]. Another strategy is to lower rates of chromosome instability by reducing high rates of centrosome amplification; this has been accomplished by the downregulation of TTK in Her2 + breast cancer cells [32]. As cancer cells often exhibit higher rates of mitotic division and genomic instability, targeting TTK kinases can be a strategy to selectively disrupt the mitotic process in cancer cells while sparing normal cells [92].

Given its role in the development of cancers, TTK has gained significant attention as a potential clinical therapy target, including breast cancer. Moreover, TTK is known to be overexpressed in breast cancer cell lines, especially in HER2^+^ and TNBC, when compared to other subtypes and normal tissues [16, 17]. King et al. 2018 found that increased expression of TTK mRNA correlated with TNBC status and worse overall survival in patients with breast cancer [5]. Rivera-Rivera et al., using a novel breast cancer tissue microarray, found that the high expression of TTK in breast tumors correlated with high proliferation (marked with Ki-67) and mesenchymal state (low E-cadherin and high Vimentin levels) [6].

Several small-molecule inhibitors of TTK have been developed and are being evaluated in clinical trials as potential anticancer drugs [4]. These inhibitors aim to induce chromosome missegregation in cancer cells, ultimately leading to cancer cell death. Previous studies have demonstrated reduced cell proliferation and attenuation of EMT of TNBC cell lines upon TTK inhibition, suggesting that TTK inhibition can specifically suppress early stages of metastasis [5]. Moreover, selective TTK inhibitors have been shown to inhibit tumor growth in vivo models and improve overall survival with little to no toxicity when combined with docetaxel in animal models of breast cancer [10, 91]. A dose-escalation, single-agent Phase I clinical trial using the TTK inhibitor S81694 (ISRCTN35641359) showed promising results [93]. This study evaluated 38 patients with metastatic solid tumors. One patient with renal cancer had a complete response, a patient with hepatocellular cancer had a reduction in the number of lesions, and 13 remained with stable disease. The most common adverse effects were fatigue, anemia, and nausea. The most severe hematologic adverse effects were Grade 3 anemia and neutropenia at the higher dose. Another randomized Phase I dose-escalation study used BAY1217389 TTK inhibitor in combination with paclitaxel (NCT02366949) [94]. This study evaluated 64 patients with solid tumors, including TNBC, of which 31.6% achieved a partial response, 46.7% had stable disease, and 21.7% had progressive disease. The major adverse effects at the higher doses of TTK inhibitor were neutropenia nausea, fatigue, and diarrhea. Nonetheless, TTK inhibitors are showing promise in clinical trials. Specifically, BOS172722 (a selective TTK inhibitor) induces significant sensitization to cell death, particularly in highly proliferative TNBC cell lines. When used with paclitaxel, it is thought to synergize and induce chromosomal segregation defects, thus impairing cancer cell proliferation [95]. In addition, preclinical studies with a selective TTK inhibitor (CFI-402,257) showed decreased proliferation of ER^+^/HER2^−^ cell lines and suppressed tumor growth in patient-derived xenografts [96]. Most recently, a randomized Phase I trial (NCT02792465), which evaluated advanced solid tumors, including ER^+^/HER2^−^ breast cancer, showed clinical benefit rates of 12% and 25% in the CFI-402,257 alone arm and combined with fulvestrant, respectively [96]. Hence, suggesting a possible treatment of ER^+^HER2^−^ advanced breast cancer patients [97].

### PLK1

Mammalian Polo-kinase 1 (PLK1) is a serine/threonine kinase that is highly conserved from yeast to humans, as it is an important kinase for cell cycle regulation [98, 99]. PLK1 levels build up before the nucleus breaks down and peak during the G2/M phase transition, facilitating the cell’s entry into mitosis [30]. Various upstream kinases and phosphatases regulate the phosphorylation-dependent activation of PLK1. PLK1 actively regulates the proper segregation of chromosomes during mitosis and participates in phosphorylating various proteins that control the attachment and movement of chromosomes.

PLK1 plays a role in regulating centrosome and mitotic events: the maturation of the centrosome, the separation of centrosomes at G2, the bipolar spindle formation, the segregation of sister chromatids, and mitotic exit [100–103]. Thus, this kinase is responsible for maintaining genome stability during mitosis and DNA damage response [104]. PLK1 has two polo-box domains (PBDs) in the N-terminal and a kinase domain (KD) in the C-terminal [105]. Overexpression of PLK1 has been closely associated with poor prognosis and survival, as this has been seen in different types of cancer like breast, lung, ovarian carcinoma, and others [104].

Because PLK1 plays a crucial role in chromosome segregation during mitosis its overexpression can lead to errors in chromosome separation and distribution, resulting in aneuploidy and genomic instability [31]. Overexpression of PLK1 also promotes EMT and can promote motility and invasiveness in diverse types of cancer like breast, prostate, and colorectal cancer [106, 107]. PLK1 phosphorylates various cell cycle regulators, including Cdc25 and Cyclin B1, promoting cell cycle entry and progression [108]. PLK1 cooperates with TTK to regulate the spindle assembly checkpoint, since it phosphorylates TTK to enhance its kinase activity, and phosphorylates the TTK target KNL1 to regulate the spindle assembly checkpoint [90]. PLK1 also cooperates with NEK2 to potentiate the NEK2-induced phosphorylation of beta-catenin in centrosomes, an event that is crucial to the separation of centrosomes at G2 [101, 109].

Because it regulates the centrosome cycle and mitosis, PLK1 has emerged as a potentially valuable target for therapies aimed at inhibiting proliferation [110]. Disrupting PLK1 through knockout has been observed to reduce cancer cell survival, trigger apoptosis, and enhance sensitivity to chemotherapy drugs, with minimal impact on normal cells [111–113]. Given PLK1’s functional role in cell cycling throughout tumor progression, researchers have investigated PLK1 inhibitors in numerous clinical trials as potential therapeutic agents for cancer patients [114, 115]. Some of the different inhibitors targeting PLK1 are BI2536, BI6727 (Volasertib), and TKM-080301 [98].

In a study targeting small cell lung cancer BI2536 was used in a Phase II study. Since there were no responses in the patients the study was terminated [116]. A dose-escalation trial used the PLK1 inhibitor Volesartib in combination with nintedamib in advanced solid tumors (30 patients) (NCT01022853) [117]. One of these patients (with an infiltrating breast carcinoma) achieved a complete response, a patient with a bronchioloalveolar adenocarcinoma had a partial response, and stable disease was achieved in 16 patients. Dose-limiting toxicities (grade 3) included increases in the liver enzymes alanine aminotransferase and aspartate aminotransferase [98]. Another clinical trial at Phase I was performed using TKM-080301 on patients with advanced hepatocellular carcinoma in the trial it was tolerated well by the patients but as a single agent in treatment there wasn’t a meaningful change in the tumors [118].

### NEK2

NEK2, or NIMA (Never in mitosis gene A)-related kinase 2, is a serine/threonine kinase that plays a critical role in regulating mitosis in eukaryotic cells [119]. NEK2 is named after its homology to the NIMA kinase, the first identified mitotic kinase found in the fungus *Aspergillus nidulans* [120]. NEK2 is primarily associated with the control of centrosome separation, kinetochore-spindle attachments, and spindle assembly checkpoint (SAC), all key events during cell division [18]. NEK2 kinase activity is tightly regulated throughout the cell cycle, mainly mediated by factors such as MAD1 (mitotic arrest deficient-like1), HEC1 (highly expressed in cancer 1), Sgo1 (Shugoshin-1), and PLK1 (Polo-like kinase 1) [69, 101, 120]. Dysregulation of NEK2 activity may arise from p53 mutations [121], and has been implicated in various human cancers, including breast, ovarian, cervical, lung, and colon [69]. Overexpression of NEK2 leads to centrosome amplification, while the silencing of NEK2 has been shown to reduce centrosome amplification [35, 63]. Previous studies have demonstrated that high levels of NEK2 mRNA and proteins correlate with poor prognosis in patients with breast cancer [18]. Data suggests NEK2 contributes to tumor progression by modulating cell proliferation via Ki-67 [120] and Wnt signaling [122], as well as EMT processes [119]. More specifically, in studies where NEK2 was depleted, results showed inhibition of the PP1/AKT/NF-kB signaling pathway, suggesting its involvement in the NEK2 mechanism of action [119, 120]. An increasing amount of evidence indicates that NEK2 is significantly upregulated in TNBC [123], potentially driving aggressive tumor behavior and contributing to the high risk of metastasis characteristics and resistance of this cancer subtype [122].

NEK2 has recently gained attention as a potential clinical therapy target for TNBC. NEK2’s critical role in centrosome separation and mitotic progression makes it a promising candidate for therapeutic intervention [69, 124, 125]. By targeting NEK2, researchers aim to disrupt the mitotic process, causing cell cycle arrest or cell death in TNBC cells. In a study by Xing et al. 2021, it was demonstrated that NEK2 silencing inhibited cell proliferation and induced apoptosis and cell cycle arrest in TNBC cell lines [124]. Similarly, other studies have shown that silencing NEK2 expression increases sensitivity to anticancer drugs such as paclitaxel, doxorubicin, and cisplatin [69]. Recent work from our laboratory has demonstrated that the overexpression of NEK2 imparts mesenchymal characteristics to the mammary epithelial cell line MCF10A [18]. Conversely, the silencing or the chemical inhibition of NEK2 in TNBC cells suppressed early metastasis, EMT, cell migration, and cell invasion by controlling levels of the Zeb1 and Slug transcription factors. Moreover, overexpression of NEK2 in Her2^+^ tumors harboring downregulated E2F3 reduced E-cadherin levels [34]. 

Several groups have reported inhibitors against NEK2. One of these groups developed T-1101 tosylate NEK2/Hec1 inhibitor [69]. This compound suppresses tumor growth in xenograft models of liver and breast cancer (including luminal and TNBC). While the clinical translation of NEK2-targeted therapies is still in its early stages, their development represents a potential breakthrough in improving the prognosis for TNBC patients. Future research and clinical trials are crucial in determining the safety, efficacy, and suitability of NEK2 as a clinical therapy target or in combination with the standard of care for TNBC, offering new hope for patients facing limited treatment options in this aggressive breast cancer subtype.

## Conclusion

Mitotic kinases are essential proteins for cell division. However, when overexpressed, they can promote and exacerbate cell proliferation by activating multiple pathways that are associated with EMT, chromosome instability, and chemoresistance. For instance, overexpression of most mitotic kinases can activate different EMT biomarkers associated with metastatic cancer, such as TNBC. Although TNBC is considered the most aggressive subtype of breast cancer, there are no known effective targeted therapies to date. For this reason, multiple mitotic kinase inhibitors are currently being studied as potential targets to develop novel therapies that could increase life expectancy and the patient’s quality of life. Nonetheless, mitotic inhibitors are currently facing different challenges in clinical trials since high levels of toxicity have been identified. As such, most of these studies are now evaluating combination therapy at lower levels of each drug to decrease the cancer’s aggressiveness while controlling toxicity levels. Recent studies highlight the potential of combinatorial therapy using mitotic kinase inhibitors, especially for TNBC, since no current biological treatments are approved against this harmful disease. Future research and clinical trials are crucial in determining mitotic kinases’ safety, efficacy, and suitability as a clinical therapy target or in combination with standard of care for TNBC, offering new hope for patients facing limited treatment options in this aggressive breast cancer subtype.

## Data Availability

No datasets were generated or analysed during the current study.

## Abbreviations

TNBC: Triple-negative breast cancer
ER: Estrogen Receptor
PR: Progesterone Receptor
HER2: Human epidermal growth factor receptor 2
AURKA: Aurora Kinase A
AURKB: Aurora Kinase B
NEK2: Never in mitosis gene A-related kinase 2
TTK: Threonine-Tyrosine kinase
PLK1: Polo-kinase 1
EMT: Epidermal-to-mesenchymal transition
APC/C: Anaphase-promoting complex/cyclosome
H3: Histone 3
ATP: Adenosine triphosphate
SAC: Spindle assembly checkpoint
MCC: Mitotic checkpoint complex
FDA: Food and Drug Administration
KD: Kinase domain
MAD1: Mitotic arrest deficient-like1
HEC1: Highly expressed in cancer 1
Sgo1: Shugoshin-1
